# Multimodal training protocols on unstable rather than stable surfaces better improve dynamic balance ability in older adults

**DOI:** 10.1186/s11556-024-00353-8

**Published:** 2024-07-12

**Authors:** Alex Rizzato, Matteo Bozzato, Luca Rotundo, Giuseppe Zullo, Giuseppe De Vito, Antonio Paoli, Giuseppe Marcolin

**Affiliations:** 1 Department of Biomedical Sciences, University of Padova, Via Marzolo, 3, Padova, 35131 Italy; 2 Department of Industrial Engineering, University of Padova, Via Venezia, 1, Padova, 35131 Italy

**Keywords:** Aging, Balance control, External perturbation, Strength, Proprioception

## Abstract

**Background:**

There has been growing interest in using unstable devices in training protocols. This study aimed to assess the effectiveness of two multimodal exercise interventions (i.e., on stable and unstable surfaces) on dynamic balance control and lower limb strength in older adults.

**Methods:**

Sixty-two older adults were randomly assigned to two intervention groups (*N* = 20, stable group; *N *= 19, unstable group), and to a control group (*N* = 18). In this single-blinded randomized controlled study, the two intervention groups underwent a 12-week training program twice a week for 45 min, consisting of strength and balance exercises. The stable (ST) group performed the training program over stable surfaces, while the unstable (UNST) group over unstable surfaces. Dynamic balance was assessed by computing the center of pressure (CoP) trajectory while a driven movable platform induced an unexpected perturbation of the base of support. Specifically, we considered the following CoP-related parameters within a 2.5-s temporal window from the beginning of the perturbation: displacement (Area95), mean velocity (Unit Path), anterior–posterior first peak (FP), post perturbation variability (PPV), and maximal oscillations (ΔCoPMax). The dominant quadriceps strength was measured through an isometric maximal voluntary contraction on an instrumented chair.

**Results:**

Four out of five CoP-related parameters (i.e., Area95, Unit Path, ΔCoPMax, and PPV) significantly improved in the UNST group from a minimum of 14.28% (*d* = 0.44) to a maximum of 52.82% (*d* = 0.58). The ST group significantly improved only in two (i.e., ΔCoPMax, and PPV) out of five CoP-related parameters with an enhancement of 12.48% (*d* = 0.68) and 19.10% (*d* = 1.06). Both intervention groups increased the maximal isometric quadriceps strength (UNST:17.27%, *d* = 0.69; ST:22.29%, *d* = 0.98). The control group did not show changes in any of the parameters considered.

**Conclusions:**

Stable surfaces promoted faster increments of muscular strength. Unstable surfaces were more effective in enhancing dynamic balance efficiency. These findings suggested the employment of multimodal training on unstable rather than stable surfaces to potentially lower the incidence of falls in older adults.

**Trial registration:**

NCT 05769361, retrospectively registered 13 March 2023, https://clinicaltrials.gov/study/NCT05769361?lat=45.3661864&lng=11.8209139&locStr=Padova,%20Italy&distance=50&page=11&rank=107.

## Introduction

Falls are a multifactorial phenomenon representing one of the major clinical problems in older adults. Indeed, falls are a cause of increasing rates of mortality and morbidity and are significant contributors to disability or early institutionalization [1]. In 2021, the World Health Organization estimated that each year, about 684,000 individuals die from falls globally and that 37.3 million falls are severe enough to require medical attention [2]. Several factors may predict the risk of falling, including age, visual disorders, cognitive impairment, Parkinson’s disease, vitamin D deficiency, poor nutrition, psychoactive medications, cardiac arrhythmia, and stroke [3]. An age-dependent decrease in postural balance control and a progressive loss of lower limb muscle strength have been addressed as crucial causes of the risk of falling [4]. A growing amount of evidence has contended that a multi-disciplinary approach is required to lower the incidence and consequences of falls, also outside geriatric contexts [5].

Nowadays, there is consistent evidence that falls in older adults can be prevented with appropriately tailored exercise programs. Indeed, physical exercise has demonstrated its beneficial effects in reducing frailty [6], the number of falls [7], improving cognitive function, and balance [8], gait [9], and poor muscular power and functional capacity [10]. A recent meta-analysis [11] considered 88 randomized controlled trials on physical activity as the only intervention for fall-risk prevention in older adults (aged > 65). The authors found that, in community settings, physical activity practice of at least 3 h a week reduced the fall risk to 39%. Similarly, Cadore and colleagues showed that a multi-component exercise intervention (i.e., strength, aerobic, and balance exercises) reduced the fall risk up to 40% in frail older adults [7].

A review reported that fall risk was more tightly associated with dynamic than static conditions [1]. Thus, the ability to regain balance after a sudden unexpected perturbation or rapidly increase the base of support through a stepping behavior appeared essential to avoid falls [12]. Moreover, despite the age-related biological impairments, older adults could develop adaptive mechanisms in responding to sudden perturbations [13]. Hence, exercise intervention to improve balance reactive strategies was considered effective for reducing falls and improving functional outcomes [14]. Recently, due to their practical benefits and widespread applications, there has been growing interest in using unstable devices in training protocols [15]. These devices (e.g., gymnastic balls and balance discs) stimulate the postural control systems, providing an unstable base of support and requiring continuous adaptations to maintain balance [16].

To the best of our knowledge, only one study compared an exercise intervention on dynamic balance with unstable devices (e.g., soft mats and balance cushions) to a resistance training machine protocol in older adults [14]. However, the balance exercise protocol also included strength exercises. Thus, the authors administered a multimodal rather than a pure balance training. Indeed, strength exercises on resistance training machines have already demonstrated a positive effect on stability performance [17]. Moreover, studying a simulated fall from a static forward-leaning position suffered from a proper ecological approach because it never occurs in daily living situations [14]. In this regard, one of the best experimental methods to study dynamic balance is the translation of the base of support [18], particularly because approximately 50% of falls in older adults are caused by the sudden motion of the base of support [19].

Therefore, this study aimed to evaluate the effectiveness of two multimodal exercise interventions over stable or unstable surfaces on dynamic balance and lower-limb strength in older adults compared to a control group that received no intervention. The primary outcomes were the measure of the dynamic balance performance following unexpected perturbations of the base of support and the measure of the isometric lower-limb strength. The secondary outcomes were the assessment of functional mobility and gait speed in two walking tests. We hypothesized that the intervention on unstable surfaces would superiorly improve dynamic balance performance while the intervention on stable ground would produce the greatest enhancement in lower limb strength. Moreover, we hypothesized that dynamic balance and lower-limb strength improvements could enhance subjects’ functional mobility and walking speed.

## Materials and methods

### Subjects

**Fig. 1:**
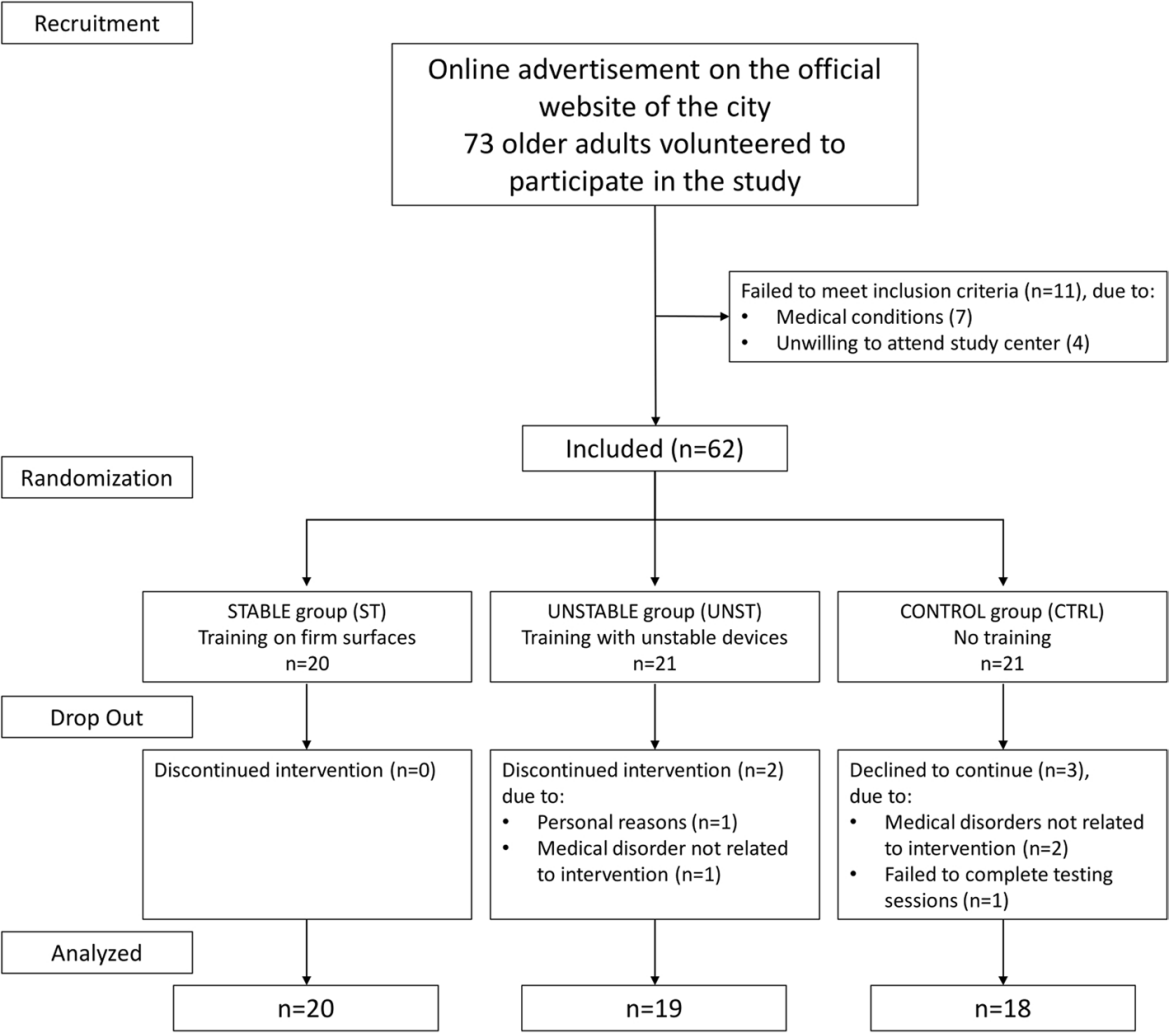
Flow diagram of the study participants

After an online advertisement, seventy-three older adults volunteered to participate in this single-blind randomized controlled study. At the time of recruitment, the subjects were included whether they were between 65–85 years old and autonomous in activities of daily living. Moreover, the following exclusion criteria were considered: (i) non-corrected sight disorders, (ii) neurological disorders, (iii) regular assumption of drugs that can interfere with normal cognitive functioning (e.g., antidepressants, antipsychotics, anxiolytics, assumption of psychotropic drugs), and (iv) other pathologies that contraindicate physical activity practice. Before enrolment, all the subjects were screened through a telephone interview according to inclusion and exclusion criteria. Eleven out of seventy-three were not eligible for the study. Thus, sixty-two older adults were enrolled in the study. For further inclusion in data analysis, subjects had to complete at least 21 out of 24 training sessions. Finally, fifty-seven subjects completed the whole experiment (Fig. 1). Three subjects discontinued participation in the study for medical disorders unrelated to the intervention; one did not complete the testing sessions, and one withdrew for personal reasons.

### Study design

The experimental protocol received approval by the ethical committee and adhered to the principles of the Declaration of Helsinki. All the subjects, after being informed about the methods of the study, gave their written informed consent and were free to renounce the study at any stage. A researcher not involved in the study and unaware of the aims randomly assigned subjects to two intervention groups (i.e., involved in the exercise program) and a control group (CTRL) with no exercise intervention. A block randomization was used for subjects’ allocation.

The stable (ST) group performed the training program over stable surfaces while the unstable (UNST) group over unstable surfaces. Furthermore, subjects were blinded to the differences between the two training programs.

**Fig. 2:**
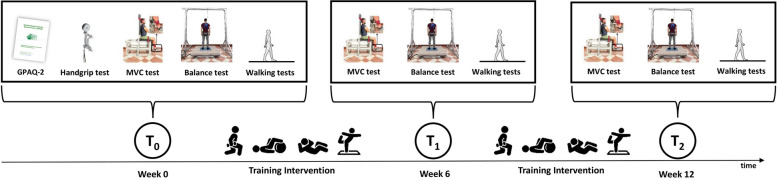
Overview of the experimental design. T_0_, T_1_ and T_2_ represent the time points of the assessments within the 12-week interventions

In this single-blinded randomized controlled study, both intervention groups received 24 training sessions of 45 min each, twice a week for 12 weeks. The program was administered in the same gym for the ST and UNST groups. The three groups were tested at the baseline (T_0_), after six weeks (T_1_), and after twelve weeks (T_2_). Subjects participated in an evaluation session consisting of functional, dynamic balance, and isometric strength assessments. The experimental design is sketched in Fig. 2.

### Training program

The training intervention was exclusively administered to ST and UNST groups, while CTRL did not receive any intervention. The training sessions were scheduled on two non-consecutive days within the same week. Subjects were randomly assigned to one out of four sports scientists involved in the study, who trained the subjects for the whole training program. Moreover, within training sessions, each sports scientist trained a maximum of two subjects. Each training session started with a 10-min warm-up, including joint mobility exercises of the lower and upper limbs (e.g., twisting/tilting of the trunk), walking gaits (e.g., on toes, on heels, heel-toe walking), and preliminary strength exercises (e.g., throwing, lifting, or moving lightweights). At the end of each session, a 5-min cool down was performed with stretching exercises. Warm-up and cool-down were standardized for both intervention groups. The 30-min central part of the training was differentiated between the ST (Table 1) and UNST (Table 2) groups, and the exercises were divided into two sessions within the same week. In detail, on the first day (training A for ST group and training C for UNST group) of the week, the following exercises were performed: hip abduction, hip flexion, balance exercise, crunch, and squat. Then, on the second day (training B for ST group and training D for UNST group) of the week, leg press, hip adduction, hip extension, calf, and supine bridge were performed. Both ST and UNST groups exercised the lower limb muscles (i.e., hip, knee, and ankle muscle groups) using mainly resistance from a Leg press machine (Technogym, Cesena, Italy), Thera Bands® (Akron, OH, United States) with different stiffness, and ankle braces (XMB002, Chinesport, Udine, Italy) with different loads. The UNST group exercised using different unstable grounds that were introduced as unpredictable disturbances: foam pad (Airex®, Sins, Switzerland), gymnastic balls (Ledragomma Srl, Osoppo, Italy) of different sizes, and balance discs (Ledragomma Srl, Osoppo, Italy). All the subjects exercised with the intensity gradually progressing over the 12 weeks using training levels of increasing difficulty (Tables 1 and 2). Subjects were asked to perform three sets of 10 to 15 repetitions for each exercise at a self-determined “moderate” intensity (i.e., 12/14 out of 20) according to the Borg Scale [3]. Subjects were questioned every session, and the trigger for progression was a perceived exertion of 8/10 out of 20 (i.e., “somewhat light”). Hence, except for balance exercises with a fixed progression, the sports scientist gradually adjusted intensity as subjects’ abilities changed throughout the program.

**Table 1:** Training exercises and volume of the group training over stable surfaces (ST)

STABLE GROUP (ST)					
**Training A**					
**Exercise**	**Training volume** **(series x reps)**	**Weeks 1–3**	**Weeks 4–6**	**Weeks 7–9**	**Weeks 10–12**
Standing hip abduction	3 × 10–15	No weights	EB	↑ EB resistance	↑ EB resistance no support
Hip flexion	3 × 10–15	Seated No weights	Seated AB (2 kg)	Standing AB (2 kg)	Standing AB (3 kg)
Monopodalic stance	3 × 30 s	No support	↑ volume (3 × 45 s)	Dual-task^a^	Dual-task^b^
Crunch	3 × 10–15	Knee-touch	Hands behind head	Hands behind head ↑ volume	Hands behind head ↑ volume
Squat	3 × 10–15	/	Half squat	Box squat	Squat
**Training B**					
**Exercise**	**Training volume** **(series x reps)**	**Weeks 1–3**	**Weeks 4–6**	**Weeks 7–9**	**Weeks 10–12**
Leg press	3 × 10–15	40% body mass	↑ load	↑ load	↑ load
Standing hip adduction	3 × 10–15	No weights	EB	↑ EB resistance	↑ EB resistance no support
Standing hip extension	3 × 10–15	No weights	AB (2 kg)	AB (3 kg)	AB (3 kg) no support
Calf	3 × 10–15	Bipodalic	Bipodalic no support	Monopodalic	Monopodalic no support
Bridge	3 × 30-45 s	/	Isometric	↑ volume	Dynamic (3 × 10–15 reps)

*EB* elastic band, *AB* ankle brace

^a^Arms abducted holding a ball (2 kg) in a static position

^b^Upper limbs movements passing a ball from one hand to the other

**Table 2:** Training exercises and volume of the group training over unstable surfaces (UNST)

UNSTABLE GROUP (UNST)					
**Training C**					
**Exercise**	**Training volume** **(series x reps)**	**Weeks 1–3**	**Weeks 4–6**	**Weeks 7–9**	**Weeks 10–12**
Standing hip abduction	3 × 10–15	On foam pad	On foam pad + EB	On balance disc ↑ EB resistance	On balance disc + EB no support
Hip flexion	3 × 10–15	On gymnastic ball	On gymnastic ball + AB (2 kg)	On gymnastic ball + AB (3 kg)	On gymnastic ball + AB (3 kg) no support
Monopodalic stance	3 × 30-45 s	On foam pad	On balance disc	On balance disc ↑ volume	On balance disc no support
Crunch	3 × 10–15	On gymnastic ball hands on chest	On gymnastic ball hands behind head	On gymnastic ball ↑ volume	On gymnastic ball ↑ volume
Squat	3 × 10–15	/	On foam pad half squat	On foam pad box squat	On balance disc box squat
**Training D**					
**Exercise**	**Training volume** **(series x reps)**	**Weeks 1–3**	**Weeks 4–6**	**Weeks 7–9**	**Weeks 10–12**
Leg press	3 × 10–15	On foam pad 40% body mass	On foam pad ↑ load	On balance disc ↑ load	On balance disc ↑ load
Standing hip adduction	3 × 10–15	On foam pad no weights	On foam pad + EB	On balance disc ↑ EB resistance	On balance disc + EB no support
Standing hip extension	3 × 10–15	On foam pad no weights	On foam pad + AB (2 kg)	On balance disc + AB (3 kg)	On balance disc + AB (3 kg) no support
Bipodalic calf	3 × 10–15	On foam pad	On foam pad no support	On balance disc	On balance disc no support
Bridge	3 × 30-45 s	/	On foam pad isometric	On foam pad ↑ volume	Dynamic on balance disc (3 × 10-15 reps)

*EB* elastic band, *AB* ankle brace

### Measurements

#### Global Physical Activity Questionnaire (GPAQ-2)

At the beginning of the study (T_0_), the GPAQ-2 was administered to estimate the daily physical activity level [20]. With 16 items, GPAQ-2 covers several physical activity components: intensity, duration, and frequency. It assesses the three domains in which physical activity is performed: (i) occupational physical activity, (ii) transport-related physical activity, and (iii) physical activity during discretionary or leisure time. The sum of the total Metabolic Equivalent (MET) minutes/week of activity was computed for a typical week in each domain.

#### Dynamic postural balance assessment

Dynamic balance control was assessed with an innovative, electrically driven movable platform (EnginLAB s.r.l., Padova, Italy) already presented elsewhere [18]. The movable platform, controlled via software (RTC-9000, EnginLAB s.r.l., Padova, Italy), allowed programming an unexpected perturbation of the base of support, acting on the displacement and the ramp rate of the platform. For the present study, the displacement was set to 50 mm, and the ramp rate was 100 mm/s. The direction of the motion was forward with respect to the standing position of the subject. A force platform (AMTI BP400600, Watertown, MA, USA) was screwed over the movable plate to calculate the Center of Pressure (CoP) trajectory during the perturbations. The CoP displacement, derived from force platforms, is considered the most reliable output for postural balance control assessment [21, 22]. The software Balance Clinic 1.4.2 allowed real-time visualization of the CoP trajectory. The sampling frequency of the force platform was set to 200 Hz. An external trigger synchronized the force platform and the movable platform. Each trial lasted 60 s, and between the twentieth and the fortieth-second, the operator randomly administered the unexpected perturbation. Out of the five trials, two no-perturbation trials were randomly administered to prevent the subject from thinking that the perturbation would have always occurred. All subjects wore a safety harness attached to an overhead frame to prevent falling in case of loss of balance due to unexpected plate shifting [18]. The safety system did not affect the posture of the subjects and let them move without any constraints in response to the sudden perturbation. For each trial, subjects were asked to stand over the movable system with arms along their sides and knees extended, gazing at a reference in front of them at 0.80 m. The feet position was recorded at T_0_ to replicate the same position over the force platform in the following testing sessions (i.e., T_1_, T_2_).

#### Upper and lower limb strength assessment

At the beginning of the study (T_0_), the grip strength of the dominant hand was measured using a handgrip dynamometer (Jamar, JLW Instruments, Chicago, IL, USA) [23]. Three consecutive measurements were made with subjects seated upright and elbow flexed at 90°. The dominant lower limb strength was evaluated in the three testing sessions through an isometric maximal voluntary contraction (MVC) of the quadriceps [24, 25]. The experimental setup consisted of a custom-built chair instrumented with a uni-axial load cell (MuscleLab, Ergotest Innovation, Stathelle, Norway) positioned three centimeters above the malleolus. The subjects performed the MVC seated with the knee flexed at 90 degrees and secured to the chair with straps to minimize additional body movements. Subjects were asked to keep their hands crossed over the chest for the whole test duration. Before the test, ten submaximal contractions were performed as a warm-up. Then, three maximal trials with real-time feedback of the actual force were performed with 40 s of recovery in between. Each MVC lasted 3 s, during which the operator verbally prompted the subject to achieve the maximal effort.

#### Walking tests

In the Timed Up and Go (TUG) test, subjects were required to stand up from the chair, walk at the preferred pace to a cone at 3 m, turn around, and walk back to the chair to sit down [26]. The timing started at the word “Go” by the operator and stopped after the subject sat on the chair. In the 10-m walking test, subjects were instructed to walk 20 m at their preferred speed. The timing started when the subjects passed the 5-m line and stopped when they crossed the 15-m line away on the floor from the starting position. In both walking tests, three trials were performed with 30 s of recovery.

### Data analysis

**Fig. 3:**
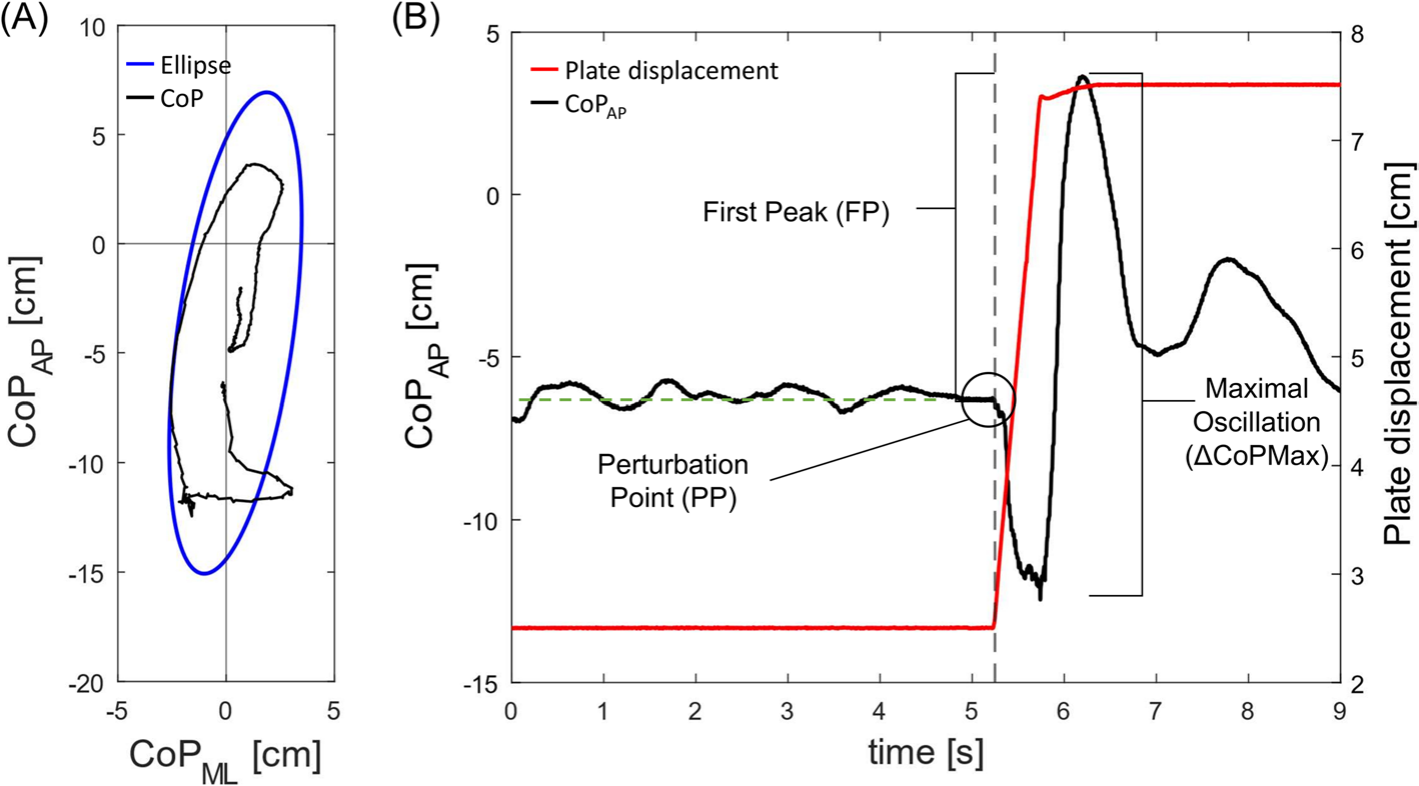
Graphical representation of the center-of-pressure (CoP) parameters referred to the dynamic balance assessment over the electrically-driven mobile platform. **A** CoP trajectory (black line) and 95^th^ percentile ellipse (blue line) within the 2.5-s time window from the beginning of the perturbation. **B** CoP-related parameters referred to the anterior–posterior CoP trajectory (black line) following the electrically-driven mobile platform displacement (red line). The gray dotted line marks the perturbation point (PP); the green dotted line represents the mean value of the anterior–posterior CoP trajectory before the perturbation occurs

In the present study, the calculation of CoP parameters in the dynamic balance tests followed the procedure already presented elsewhere [18]. The perturbation point (PP) was identified as the instant the movable platform moved. The Unit Path (the mean velocity measured in cm∙s^−1^) and Area95 (the area of the 95th percentile confidence ellipse measured in cm^2^) were calculated over a 2.5-s time window after the PP. Moreover, we calculated three additional parameters (Fig. 3) to deepen the postural responses in the direction of the perturbation (i.e., posterior-anterior). The first peak (FP) represents the difference between the maximal peak reached by the anterior–posterior CoP trajectory after the PP and its mean value before the PP. The maximal oscillation (ΔCoPMax) was calculated as the sum of the absolute values of FP and the subsequent peak. The post-perturbation variability (PPV) was defined as the standard deviation (SD) of the anterior–posterior CoP trajectory over the 2.5-s time window after the PP, and it is an index of the efficiency of the subject in reducing the body oscillations immediately after the external perturbation to reach a new quiet condition [18].

The handgrip test measured the isometric grip strength of the dominant hand in kilograms. On the other hand, the maximum strength of the dominant lower limb was expressed in Newton and normalized with respect to the subject’s body mass (% BM).

### Statistical analysis

The a-priori power analysis calculation (G * Power 3.1.9.2 software) showed that a total sample size of 51 participants and a medium effect size (f) of 0.25 would have provided a statistical power of 0.8 with an α error probability of 0.01. The enrolment of sixty-two adults allowed the required sample size to be achieved despite five dropouts. The dropout subjects were excluded from the study and thus, were not considered in the statistical analysis. The mean value among the three trials was calculated in the dynamic balance and walking tests. Conversely, the highest value of the three trials was considered in the upper and lower limb strength. Data are presented as mean and standard error of the mean (SE). The Shapiro–Wilk test checked the normality distribution of data. The one-way analysis of variance (ANOVA) compared MET data, handgrip values, and groups’ characteristics at the baseline (T_0_). Then, a two-way mixed-model ANOVA for repeated measures investigated the main effects of training (i.e., T_0_, T_1_, and T_2_), groups (i.e., ST, UNST, and CTRL), or any interactions. In case of a statistically significant main effect or interaction, the Holm-Bonferroni post hoc test assessed the pairwise comparisons. The effect size (*d*) was calculated to assess the strength of potential changes following the exercise interventions (T_0_ vs. T_2_) for all variables. The magnitude of the effect size was interpreted as follows: partial eta-squared (ηp^2^): small ≥ 0.01, medium ≥ 0.06, and large ≥ 0.14 [27]; Cohen’s *d*: small (0.2 ≤ *d* < 0.5), medium (0.5 ≤ *d* < 0.8), and large (*d* ≥ 0.8) [28]. The significant level for differences was set to *p* < 0.01. JASP Software (University of Amsterdam, Amsterdam, The Netherlands), version 0.16.4.0, was used for statistical analysis.

## Results

Characteristics of all groups are reported in Table 3. The one-way ANOVA for baseline comparisons (T_0_) showed no statistically significant differences among groups for MET (*F* = 0.355; *p* = 0.703; ηp^2^ = 0.013. UNST: 2822.10 ± 503.03; ST: 2187.00 ± 598.36; CTRL: 2394.44 ± 530.20), and handgrip values (*F* = 0.529; *p* = 0.592; ηp^2^ = 0.019. UNST: 33.07 ± 2.42 kg; ST: 30.02 ± 1.63 kg; CTRL: 31.11 ± 2.37 kg). Subjects did not report any adverse effects during the study. Moreover, the adherence to the training was similar in the two intervention groups: ST 95.83% (23.00 ± 0.22 training sessions) and UNST 96.25% (23.11 ± 0.20 training sessions). The results of dynamic balance, strength, and functional tests are summarized in Table 4. Moreover, Table 5 reported the changes in percentage and Cohen’s *d* of the outcome parameters before and after the 12-week training interventions.

**Table 3:** Participants’ characteristics

	**Subjects**	**Age (yrs)**	**Height (m)**	**Mass (kg)**
**ST**	20 (M = 6)	74.30 ± 1.12	1.64 ± 0.02	70.63 ± 2.86
**UNST**	19 (M = 9)	69.74 ± 0.86	1.65 ± 0.02	70.87 ± 3.26
**CTRL**	18 (M = 8)	72.44 ± 1.36	1.66 ± 0.03	67.14 ± 2.70

Data are presented as mean and standard error of the mean (SE)

*ST* stable group training over stable surfaces, *UNST* unstable group training over unstable surfaces, *CTRL* control group, no training, *M* male

**Table 4:** Outcome parameters before (T_0_), at six weeks (T_1_) and after (T_2_) the intervention for the three groups. Data are presented as mean and standard error of the mean (SE)

	**ST**			**UNST**			**CTRL**		
	T_0_	T_1_	T_2_	T_0_	T_1_	T_2_	T_0_	T_1_	T_2_
**Unit Path (cm·s**^**−1**^) ^*, †^ **(95% CI)**	23.53 ± 1.03 (21.39,25.68)	22.67 ± 0.93 (20.72,24.62)	23.02 ± 0.90 (21.14,24.91)	27.10 ± 2.29 (22.29,31.90)	23.99 ± 1.85 (20.10,27.88)	23.22 ± 1.12^**‡**^ (20.86,25.59)	24.49 ± 1.10 (22.15,26.83)	26.11 ± 1.60 (22.72,29.51)	23.40 ± 1.01 (21.25,25.55)
**Area95 (cm**^**2**^) ^*, †^ **(95% CI)**	55.66 ± 10.31 (34.07,77.24)	39.06 ± 6.91 (24.60,53.52)	39.16 ± 5.53 (27.59,50.73)	98.37 ± 23.14 (49.75,147.0)	56.68 ± 18.70^**‡**^ (17.39,95.97)	46.41 ± 7.97^**‡**^ (29.66,63.16)	52.87 ± 9.72 (32.26,73.49)	64.02 ± 15.29 (31.60,96.44)	33.86 ± 3.81 (25.78,41.93)
**First Peak (cm) **^**†**^ **(95% CI)**	6.96 ± 0.18 (6.59,7.34)	6.67 ± 0.17 (6.32,7.02)	7.01 ± 0.18 (6.65,7.38)	7.25 ± 0.14 (6.96,7.55)	7.03 ± 0.21 (6.60,7.46)	6.72 ± 0.19 (6.32,7.11)	7.08 ± 0.22 (6.60,7.55)	7.43 ± 0.23 (6.93,7.92)	7.30 ± 0.22 (6.82,7.77)
ΔCoPMax (cm) ^*, †^ **(95% CI)**	12.07 ± 0.52 (10.99,13.16)	9.82 ± 0.39^**‡**^ (9.01,10.63)	10.57 ± 0.46^**‡**^ (9.61,11.52)	12.62 ± 0.53 (11.51,13.74)	10.99 ± 0.62^**‡**^ (9.69,12.29)	9.95 ± 0.55^**‡**^ (8.79,11.10)	11.37 ± 0.58 (10.14,12.60)	11.37 ± 0.56 (10.19,12.56)	10.66 ± 0.51 (9.57,11.74)
PPV (cm) ^*, †^ **(95% CI)**	3.00 ± 0.13 (2.72,3.27)	2.31 ± 0.07^**‡**^ (2.16,2.46)	2.42 ± 0.11^**‡**^ (2.20,2.65)	3.21 ± 0.17 (2.85,3.56)	2.61 ± 0.20^**‡**^ (2.18,3.04)	2.38 ± 0.14^**‡**^ (2.09,2.67)	2.78 ± 0.16 (2.43,3.12)	2.62 ± 0.16 (2.28,2.96)	2.36 ± 0.12 (2.11,2.61)
Strength (%BM) ^*, †^ **(95% CI)**	56.49 ± 2.95 (50.29,62.68)	67.70 ± 3.38^**‡**^ (60.61,74.80)	69.08 ± 2.93^**‡**^ (62.92,75.23)	64.21 ± 2.53 (58.90,69.52)	71.24 ± 4.01 (62.63,79.85)	75.30 ± 4.20^**‡**^ (66.47,84.13)	59.60 ± 3.81 (51.43,67.77)	60.93 ± 3.27 (53.92,67.94)	59.88 ± 3.72 (51.90,67.86)
TUG (s) ^*^ **(95% CI)**	8.85 ± 0.20 (8.44,9.26)	8.90 ± 0.22 (8.43,9.36)	8.41 ± 0.17 (8.06,8.76)	9.18 ± 0.24 (8.67,9.68)	9.10 ± 0.25 (8.59,9.62)	8.65 ± 0.20 (8.23,9.06)	9.98 ± 0.43 (9.07,10.88)	9.68 ± 0.37 (8.90,10.45)	9.55 ± 0.40 (8.71,10.38)
**10-m (s)** **(95% CI)**	7.35 ± 0.18 (6.98,7.72)	7.28 ± 0.12 (7.03,7.54)	7.20 ± 0.13 (6.92,7.48)	7.54 ± 0.10 (7.32,7.76)	7.59 ± 0.15 (7.27,7.92)	7.43 ± 0.15 (7.11,7.75)	7.71 ± 0.26 (7.16,8.26)	7.62 ± 0.22 (7.15,8.08)	7.69 ± 0.26 (7.16,8.23)

^*^Statistically significant training effect (*p* < 0.01)

^†^Statistically significant training-by-group interaction (*p* < 0.01)

^‡^ Statistically significant differences (post-hoc analysis) from baseline (T_0_) (*p* < 0.01)

**Table 5:** T_0_ to T_2_ differences in percentage (Δ%) of the outcome parameters together with the correspondent effect size values (Cohen’s *d*)

	**ST**		**UNST**		**CTRL**	
	Δ% (T_2_-T_0_)	*Cohen’s d*	Δ% (T_2_-T_0_)	*Cohen’s d*	Δ% (T_2_-T_0_)	*Cohen’s d*
**Unit Path (cm·s**^**-1**^**)**	-2.16	0.19	-14.28	0.44	-4.46	0.25
**Area95 (cm**^**2**^**)**	-29.64	0.41	-52.82	0.58	-35.96	0.54
**First Peak (cm)**	0.73	0.06	-7.37	0.72	3.12	0.24
**ΔCoPMax (cm)**	-12.48	0.68	-21.21	1.10	-6.29	0.31
**PPV (cm)**	-19.10	1.06	-25.75	1.21	-14.84	0.68
**Strength (%BM)**	22.29	0.98	17.27	0.69	0.46	0.02
**TUG (s)**	-4.99	0.53	-5.78	0.54	-4.31	0.24
**10-m (s)**	-2.02	0.20	-1.39	0.17	-0.24	0.01

### Dynamic balance

At the baseline (T_0_), no statistically significant differences among groups were detected. The statistical analysis showed a significant main effect of the training (*F* = 6.023; *p* = 0.003; ηp^2^ = 0.102) and an interaction training vs. group (*F* = 4.424; *p* = 0.002; ηp^2^ = 0.143) in the Unit path. For the Area95, a significant main effect of the training (*F* = 10.492; *p* < 0.001; ηp^2^ = 0.165) and an interaction training vs. group (*F* = 3.568; *p* = 0.009; ηp^2^ = 0.119), was detected. For the FP, a non-significant training effect was observed (*F* = 0.432; *p* = 0.650; ηp^2^ = 0.008) but a significant interaction training vs. group (*F* = 4.869; *p* = 0.001; ηp^2^ = 0.155). However, post hoc comparisons did not detect any significant differences within groups. The statistical analysis showed a significant main effect of the training for both ΔCoPMax (*F* = 30.890; *p* < 0.001; ηp^2^ = 0.368) and PPV (*F* = 43.951; *p* < 0.001; ηp^2^ = 0.453). Moreover, a statistically significant interaction training vs. group was observed for both ΔCoPMax (*F* = 7.411; *p* < 0.001; ηp^2^ = 0.219) and PPV (*F* = 3.919; *p* = 0.005; ηp^2^ = 0.129).

### Strength and walking tests

Results of maximal isometric strength showed a significant main effect of the training (*F* = 20.081; *p* < 0.001; ηp^2^ = 0.287) and an interaction training vs. group (*F* = 4.420; *p* = 0.002; ηp^2^ = 0.150). For the TUG, the two-way ANOVA showed a significant main effect of training (*F* = 9.661; *p* < 0.001; ηp^2^ = 0.152), but the post hoc comparisons did not detect any significant differences within groups for TUG. Moreover, the statistical analysis highlighted a non-significant interaction between training vs. group (*F* = 0.642; *p* = 0.634; ηp^2^ = 0.023). The 10-m walking test was unaffected by either training (*F* = 0.593; *p* = 0.554; ηp^2^ = 0.011) and interaction training vs. group (*F* = 0.406; *p* = 0.804; ηp^2^ = 0.015).

## Discussion

The present study evaluated the effectiveness of two multimodal interventions in older adults, different for the surface where lower limb strength and balance exercises occurred: stable and unstable surfaces for ST and UNST groups, respectively. Indeed, poor muscle strength and dynamic balance represent notable fall-risk factors in older adults [1]; thus, the research of increasingly effective training interventions is fundamental to mitigate this public health issue. Both exercise interventions presented in our study improved lower limb strength and dynamic balance at 12 weeks. These findings are remarkable considering that physiological systems contributing to balance ability decreased with aging and could be associated with increased fall risk [29]. In detail, the training on unstable surfaces highlighted a better balance performance (i.e., lower values of Area95) and a higher efficiency of the postural control systems (i.e., lower values of Unit Path) in coping with the external perturbations superimposed by the electrically-driven movable platform. Moreover, although the dynamic balance improvements (Table 5) in both intervention groups were significantly greater compared to the control group, effect sizes were higher in the UNST (*Cohen’s d* from 0.44 to 1.21) rather than in the ST (*Cohen’s d* from 0.06 to 1.06) group. Considering that older adults demonstrated an increased risk of falling when unable to rapidly plan strategies and respond effectively to base-of-support changes [30], our findings support multimodal training on unstable surfaces as an effective choice to improve dynamic balance control in older adults. The main mechanism underpinning the overall better balance performance of the UNST group could be attributed to the stimuli the unstable surfaces gave the participants. Indeed, the induced instability could have introduced repeated changes in acting forces and unpredictable sensory inputs that highly stimulated the proprioceptive system [14]. In this regard, the training protocol on unstable surfaces could have improved the demand on the nervous system to perceive sensory signals and generate appropriate motor commands [31]. Conversely, the less striking improvements in dynamic balance control in the ST group could be attributable to the control mechanisms of the CoP displacement within the base of support that is related more to sensory perception than to muscle strength [32, 33].

Overall, in a dynamic environment, CoP-related parameters are more sensitive than functional test outputs in the balance scoring process [34], reducing the risk of not highlighting training advancements. Indeed, the employment of an electrically driven movable platform and the specific CoP-related parameters [18] represented a novelty in this longitudinal study. The FP reflects the efficacy of the earliest feet-in-place postural responses to the perturbation of the base of support and depends mainly on the spinal cord-mediated stretch reflexes with the shortest latencies (< 70 ms). The non-significant changes of FP over the 12 weeks in both ST and UNST groups could depend on the training modalities that did not include exercises with sudden unexpected perturbations. Conversely, voluntary responses have more prolonged latencies (> 150 ms) and produce highly variable motor responses [35]. Since most of the dynamic CoP-related parameters calculated (i.e., Area95, Unit Path, ΔCoPMax, and PPV) assessed postural responses with latencies longer than 150 ms, we can speculate that exercises of both interventions (Tables 2 and 3) trained mainly voluntary controlled mechanisms.

Moreover, unlike previous studies [36], the exercises in our multimodal training programs differed completely from the dynamic balance test performed over the electrically driven movable platform. Consequently, in agreement with Bierbaum and colleagues [37], our findings provided indirect evidence that both multimodal training protocols produced motor and perceptive schemes useful outside of the specific training domain. It has been argued that new postural strategies may be ascribed to a shift from prefrontal activity to a subcortical circuit, accompanied by increased automatic balance performance [38]. Considering the higher balance improvements of the UNST group, we can speculate that repetitive training with unstable devices could boost sensorimotor adaptations transferable to daily living postural control. Indeed, the repeated exercises proposed with the unstable training protocol enhanced balance skills not only within the same repeated exercises but also in other untrained demanding balance tasks (i.e., responding to a sudden perturbation of the base of support) [39]. Hence, the multimodal training protocol over unstable surfaces could supposedly help minimize the risk of falls in older adults.

The age-related reduction of muscle strength is considered per se one of the higher risk factors for falls in older adults [14]. Various strength training, from highly controlled lab-based to minimally supervised home-based programs, elicited meaningful benefits in older adults. Although the two interventions presented in this study were not fully oriented to increase strength, they showed improvements (Table 5) in the quadriceps isometric strength compared to the control group. After six weeks, the isometric strength of knee extensors significantly increased only in the ST group, which maintained this improvement until week 12. Conversely, the unstable training did not trigger a strength increase at the early stage and exhibited nearly similar increases to the stable training only at week 12. Thus, contrary to our hypothesis, both multimodal trainings led to similar enhancement in lower limb strength over the twelve weeks. However, the different training surfaces used by the two intervention groups could account for the earlier strength enhancement in the ST group. Although the perceived exertion was the same for the two groups, strength exercises of the UNST group did not allow the same load progressions over the twelve weeks compared to the ST group (mean load: ~ -20%). Indeed, given the need for continuous adaptation to unstable surfaces, exercising with unstable devices might cause reduced force production during training [40]. Overall, strength increments detected in UNST and ST were moderate compared to those following protocols oriented to resistance training, namely ~ 35% [41] and ~ 37% [42]. However, they were in line with increments of similar studies using multimodal exercise protocols on stable surfaces: ~ 20% [43], ~ 20% [10], and ~ 19% [44]. Notably, the mentioned studies were performed on highly deconditioned subjects. Indeed, institutionalized older adults, considering their functional loss, could obtain more significant functional gains following multimodal training (i.e., strength, mobility, and balance) compared to healthy, active older adults [45]. Hence, our results expanded previous findings in deconditioned subjects, demonstrating that multimodal training protocols based on balance and strength exercises also positively affected strength in active older adults.

Finally, although dynamic balance control and lower-limb isometric strength increased after training, contrary to our second hypothesis, the functional walking tests showed no improvements during and after the training interventions. Even though these tests are valid and reliable in assessing health-related physical fitness, our findings could be explained by the ceiling effect these tests presented when applied in high-functioning older adults [46].

In conclusion, the two multimodal training programs increased muscular strength and dynamic balance control at 12 weeks. The stable surfaces promoted a faster increment of muscular strength, while the unstable surfaces enhanced the mechanisms underlying dynamic balance efficiency in facing sudden perturbations of the base of support. Therefore, the use of unstable as compared with stable surfaces is more effective in improving physiological parameters related to fall risk. Future research might explain how unstable surfaces in multimodal training could target fall prevention compared to traditional approaches in community-dwelling populations or highly deconditioned older adults.

The present study has some potential limitations that need to be acknowledged. The recruited sample included male and female subjects, but it was impossible to perform statistical analyses on sex differences because of the relatively small number of subjects in each group. Then, to guarantee subjects’ safety, it was impossible to include any exercise with sudden unexpected perturbations in training protocols. Thus, the interventions stimulated mainly voluntary control mechanisms. Finally, although comparing two 12-week multimodal training protocols performed on different grounds could represent a novelty in this field, we did not assess the long-term retention of the obtained benefits.

## Data Availability

The datasets used and/or analyzed during the current study are available from the corresponding author on reasonable request.

## Abbreviations

ANOVA: Analysis of variance
BM: Body mass
CoP: Center of Pressure
CTRL: Control group
FP: First Peak
GPAQ: Global Physical Activity Questionnaire
MVC: Maximal voluntary contraction
PP: Perturbation point
PPV: Post-perturbation variability
ST: Stable surfaces
TUG: Timed Up and Go test
UNST: Unstable surfaces
ΔCoPMax: Maximal oscillation
